# Characterization of casein hydrolysates derived from enzymatic hydrolysis

**DOI:** 10.1186/1752-153X-7-62

**Published:** 2013-04-04

**Authors:** Jinshui Wang, Yinjie Su, Feng Jia, Huali Jin

**Affiliations:** 1 College of Biological Engineering, Henan University of Technology, Zhengzhou, 450001, People’s Republic of China; 2 College of Food Science and Technology, Henan University of Technology, Zhengzhou, 450001, People’s Republic of China

**Keywords:** Casein-trypsin system, Enzymatic hydrolysis, Molecular weight distribution, Secondary structure

## Abstract

**Background:**

Casein is the main proteinaceous component of milk and has made us interest due to its wide applications in the food, drug, and cosmetic industries as well as to its importance as an investigation material for elucidating essential questions regarding the protein chemistry. Enzymatic hydrolysis is an important method commonly used in the modification of protein structure in order to enhance the functional properties of proteins. The relationship between enzymatic hydrolysis and structure change of casein need to make more study.

**Results:**

During hydrolysis, degree of hydrolysis in the casein hydrolysates increased rapidly in the initial 20 minutes, reached a plateau after 45 minutes, and then kept relative constant for the rest of the hydrolysis. The relative percentage of the released peptides with molecular weight of over 50 kD significantly decreased with hydrolyzation, while those with MW of 30–50 kD and below 20 kD increased significantly. The contents of a-helix and β-turn in the hydrolysates increased compared to the original casein. Moreover, the molecular flexibilities of the casein hydrolysates, estimated by the ratio of α-helix to β-structure, were lower than that of original casein protein.

**Conclusions:**

The significant changes in molecular weight distribution and structure characteristics of casein hydrolysates were found compared to the control sample. This change should be the basis of enhancement of functional properties.

## Background

Casein is the main proteinaceous component of milk, where it accounts for about 80% of the total protein inventory. Casein has made us interest due to its numerous using in the food, drug, and cosmetic industries as well as to its importance as an investigation material for elucidating essential questions regarding the protein chemistry [1-4]. Technically, it is part of a group called phosphoproteins, collections of proteins bound to something containing phosphoric acid. Casein includes four individual gene product components denoted αs1-, αs2-, β- and κ-casein, which differ in primary structure and type and degree of post-translational modification [5]. These four casein types are essentially different in their molecular weights as follows: αs1-casein (MW = 23 KD, ~38.49%), αs2-casein (MW 25KD, ~10.06%), β-casein (MW 24KD, ~38.74%), κ-casein (MW19KD, ~12.57%) [6]. The main physiological role of casein in the milk system was widely accepted to be a source of amino acids required by growth of the neonate. However, the dominant physiological feature of the casein micelle system has more recently been proven to be the prevention of pathological calcification of the mammary gland [7]. Due to the excellent functional properties and natural abundance, casein proteins (or their hydrolysates) represent a privileged and crucial tool for the food industry. Their hydrodynamic and surface-related properties lead to suitable functionalities that are utilized for countless manufactured products [8,9]. They can also contribute to improve color and flavor of food products.

Enzymatic hydrolysis is commonly used method in the modification of protein structure in order to enhance the functional properties of proteins [10]. Since the hydrolysation of proteins makes change in the composition of potential groups and hydrophobic properties, functional characteristics are also changed. There are many studies in this filed. The enhancement of these properties should stem from the change in structure of protein. Because the structural basis of the protein hydrolysates was one of the most important factors concerning desired functional properties used as functional materials. However, little information about the structural changes of protein hydrolysates is available.

For this reason, this study was conducted to study the change in the structure of the resultanting hydrolysates during proteolysis of casein by means of fourier transform infrared (FTIR) spectroscopy. Moreover, the molecular mass distribution and free amino acid composition of casein hydrolysates were analyzed. Differences in molecular weight of these hydrolysates were compared by SDS-PAGE.

## Materials and methods

### Raw materials

Casein protein sample was purchased from Huigong Co., Zhengzhou, China. Casein contained 82.5% (w/w, dry basis) protein and 11.9% moisture. The commercial enzyme (trypsin, 1.2 × 10^5^ U/g) was purchased from Amresco, USA. The other chemicals were of analytical grade.

### Preparation of casein hydrolysates

An 8% (w/v) aqueous dispersion of casein was incubated at water bath at 40°C for 10 min. When the casein dispersion reached 40°C, the protease, trypsin at enzyme to substrate [E/S] 2500 U/g was added. Enzymatic hydrolysis was carried out at constant pH 8.0. The enzyme was inactivated for 10 min by heat treatment at 100°C. The resulting hydrolysate was then rapidly cooled to about 25°C in an ice bath, and then freeze-dried and stored at −20°C until use.

### Determination of degree of hydrolysis

The degrees of hydrolysis of gluten hydrolysates were measured by the *o*-phthaldialdehyde (OPA) method [11]. The gluten hydrolysate powder was solubilized at 1.25 mg ml^-1^, in 12.5 mM borate buffer (pH 8.5) plus 2% (w/v) SDS. This solution (50 μl) was mixed with 1 ml of the reagents. The reagent composed of 50 ml of 0.1 M borate buffer (pH 9.3), 1.25 ml of 20% (w/v) SDS solution, 100 mg of N, N-dimethyl-2-mercaptoethylammonium chloride (DMMAC) and 40 mg of OPA dissolved in 1 ml methanol. The mixture was allowed to stand for 2 min before measurement the absorbance at 340 nm. The number of amino groups was determined with reference of the L-leucine standard curve (between 0.5 and 5 mM). The increase in amino groups between native gluten and hydrolysates was attributed to proteolysis and DH was calculated by the following equation:

$$\mathrm{DH}\;\left(\%\right)=\left[\left(\alpha -n_{i}\right)/n_{T}\right]\times 100$$

where *n*_*T*_ was the total number of amino groups in native gluten after total hydrolysis with 6 M HCl for 24 hours and *n*_*i*_ was the number of amino groups in native gluten while α was the number of free amino groups measured in the gluten hydrolysate.

### Molecular mass distribution of casein hydrolysates

The molecular mass distributions of supernatants in the hydrolysates were estimated by gel permeation chromatography on Agilent PL aquagel-OH MIXED-H column (Agilent, LC1260, USA) with a UV detection at 214 nm. Elution was achieved at 0.5 ml min^-1^ by 0.25 M phosphate buffer (pH 7.2). The column was calibrated with bovine serum albumin (MW66 kDa), egg albumin (MW 44,287 Da), cytochrome C (MW 12,384 Da), aprotinin (MW 6511.44 Da), VB12 (MW 1355.37).

### Amino acid analysis

The PICO TAG method, with minor modifications, was used for measuring the amino acid profile of the hydrolysate [12]. The dry sample (weight equivalent to 4% protein) was added with 6 M HCl (15 ml) and placed in the oven at 110°C for 24 h. 10 ml of internal standard was added to the mixture. After derivatisation, 100 μl PICO TAG diluent was added and mixed. 100 μl of sample were then injected into the HPLC and analyzed with the Water’s PICO TAG amino acid analyzer.

### FTIR measurements

Fourier transform infrared (FTIR) spectra of original casein and the casein hydrolysate samples were recorded using a WQF-510 FTIR spectrometer (Beijing Beifen-Ruili Analytical Instrument (Group) Co. Ltd.) equipped with a deuterated triglycine sulphate detector. The spectrometer was continuously purged with dry air from a Balston dryer (Balston, MA). The sample powder (maintained at ambient temperature) included 2 mg sample per 200 mg of KBr. After homogenising with an agate mortar and pestle, the powder was pressed into pellets (1–2 mm thick films) with a 15-ton hydraulic press. FTIR spectra were obtained of wave number from 400 to 4000 cm^-1^ during 128 scans, with 2 cm^-1^ resolution (Paragon 1000, Perkin-Elmer, USA). Interpretation of the changes in the overlapping amide I band (1600–1700 cm^-1^) components was made possible by deconvolution using Peak-Fit v4.12 software (SPSS Inc.). And then a linear baseline between 1600 cm^-1^ and 1700 cm^-1^ was formed and the baseline was linearly corrected.

### SDS-PAGE analysis

Sodium dodecyl sulphate-polyacrylamide gel electrophoresis (SDS-PAGE) was conducted according to the method of Laemmli (1970) [13] using 15% (v/w) acrylamide separating gel and 4% acrylamide stacking gel. Samples were prepared in Tris–HCl buffer (pH 6.8) containing 2% SDS and 0.2% β-mercaptoethanal. The gel sheets were stained with Coomassie brilliant blue R-250.

### Statistical analysis

DH was determined four times while molecular weight distribution and amino acid content were measured in duplicate. Consequently, a variance analysis (ANOVA) was performed on each experiment to determine the effect of hydrolysis at 95% or 99% level.

## Results and discussion

### Time-dependent degree of casein hydrolysis

**Figure 1:**
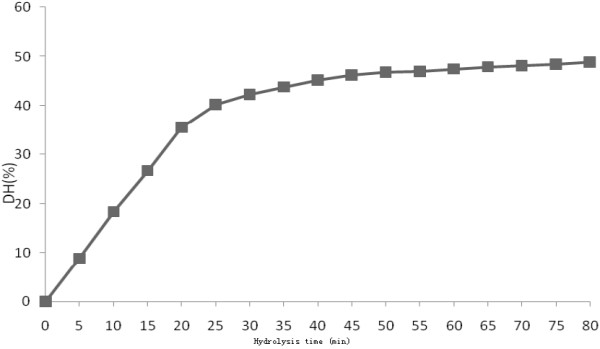
Changes in degree of hydrolysis of casein protein during enzymatic hydrolysis.

The extent of proteolysis was generally quantified as the degree of hydrolysis (DH) referred to the percentage of peptide bonds cleaved. Different methods were used to evaluate the DH of the peptide bonds which depended on three essential principles: the amount of nitrogen released by the protein hydrolysis in the presence of a precipitation agent (e.g. trichloroacetic acid), the determination of free α-amino groups and the titration of the released protons. These measurements showed the advantage during hydrolysis, but it was impossible to follow the DH when the viscosity started to increase. Thus, the OPA method was used in the present study. The presence of the SDS in the OPA solution served to inactivate the enzyme and ensured a full exposure of amino groups. Therefore, it was possible to measure the DH in our study. As shown in Figure 1, the DH increased rapidly in the first 20 minutes, due to high protein content and enzyme concentration. The DH reached a plateau after 45 minutes (DH = 45.2%) and kept relative constant for the rest of the hydrolysis reaction.

### Molecular weight distribution of the peptides released during enzymatic hydrolysis of casein protein

**Figure 2:**
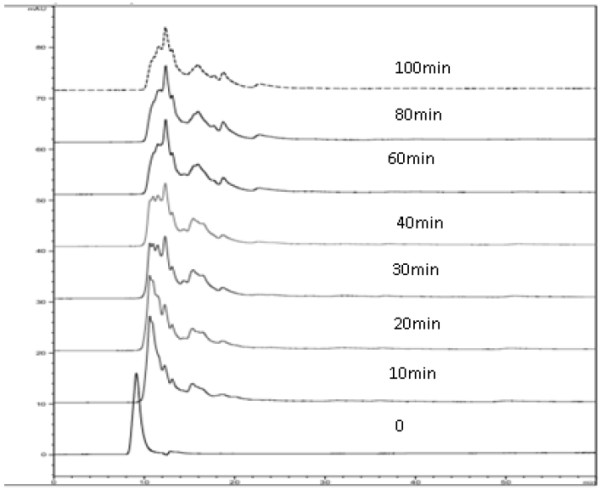
Molecular weight distriutions of hydrolysated supernatants from casein protein.

For the purpose of a more complete characterization of the proteolysis process, chromatographic separation (HPLC) of the released peptides during enzymatic hydrolysis was carried out (Figure 2). The molecular weight distribution determined by HPLC was shown in Table 1. The relative percentage of the peptides released with molecular weight (MW) of over 50 kD significantly (*P* < 0.05) decreased with increasing enzymatic hydrolyzation, while those with MW below 20 kD increased significantly (*P* < 0.05). Especially, significant changes (*P* < 0.05) in the relative percentages of these two parts of released peptides were found at 20 min of enzymatic hydrolysis compared to the control. Meanwhile, the relative percentages of the released peptides with MW of 40–50 kD, 35–40 KD and 30–35 KD rapidly rose at the initial stage of hydrolysis, and then began to decline after 20 minutes of enzymatic hydrolysis. However, the relative percentage of the peptides with MW of 20–30 kD increased initially, and then kept constant. The molecular weight of the peptides from the protein hydrolysates was one of the most important factors concerning desired functional properties used as functional materials [14] (Deeslie and Cheryan, 1991). There are totally differences in their functional properties of the casein hydrolysates with different molecular weight (It is not shown here).

**Table 1:** Relative percent of the peptides in HPLC in total area (%)

**Hydrolysis time (min)**	**Molecular weight**					
	**<20000**	**20000–30000**	**30000–35000**	**35000–40000**	**40000–50000**	**>50000**
0	4.35	1.23	0.84	1.09	3.92	88.57
10	43.13	17.20	10.33	11.22	14.68	3.43
20	50.86	17.58	8.93	8.93	11.34	2.37
30	60.33	17.96	7.19	6.5	7.44	0.58
40	63.43	17.79	6.54	5.66	6.06	0.52
50	70.46	16.90	5.38	3.92	3.19	0.14
60	71.63	16.49	5.02	3.67	3.03	0.16
80	72.46	16.01	4.74	3.54	3.08	0.17

### Change in free amino acids during enzymatic hydrolysis of casein protein

The amino acid compositions of the casein hydrolysate suspensions with different degree of hydrolysis (DH = 0, 8.9% and 26.7%) were listed in Table 2. There was no tryptophan detected for all samples because of being decomposed by HCl during hydrolysis. The content of free amino acids was lower (25.6 mg/100 ml). Obvious increases in the total contents of free amino acids in the two hydrolysates were found. The total contents of free amino acids were respectively 327.3 mg/100 ml for he hydrolysate with DH = 8.9% and 899.3 mg/100 ml for the hydrolysate with DH = 26.7%. In addition, the hydrolysates were rich in glutamic acid/glutamine, proline, and lysine. Nearly no cysine was found in the hydrolysates (Table 2). It means that the resultant peptides from casein enzymatic hydrolysis.

**Table 2:** **Amino acid composition of casein hydrolysate/mg.100 mL**^**-1**^

**Amino acid**	**Casein hydrolysate**		
	**0**	**8.9%**	**26.7%**
Asp	2.1	23.7	50.1
Glu	8.4	105.4	187.5
Ser	2.9	7.3	24.6
Gly	–	3.5	21.3
His	–	2.1	28.4
Arg	2.5	17.1	35.7
The	–	4.1	34.6
Ala	–	2.9	8.7
Pro	2.3	16.5	83.5
Tyr	0.7	5. 6	28.8
Val	1.4	47.6	64.3
Met	–	3.5	22.0
Cys	–	–	0.2
Ile	1.3	19.1	46.9
Leu	2.6	29.9	75.6
Try	1.4	24.8	55.7
Phe	–	6.8	41.3
Lys	–	7.4	90.1
Total content	25.6	327.3	899.3

### Structural characteristics of casein hydrolysates

**Figure 3:**
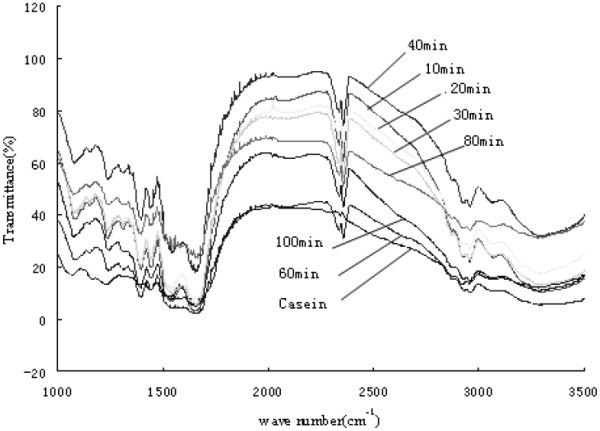
FTIR spectrum of casein and casein hydrolysates.

In the present study, fourier transform infrared (FTIR) analysis was used to investigate the difference in the secondary structures in the protein conformations (Figure 3). Amide I band was mainly due to the C = O stretching vibrations [15], which was showed by the fact that the biggest absorption peak at 1650 cm^-1^ was composed of several overlapping component bands due to the protein segments with different structures [16].

The deconvolution of amide I bands was constituted by at least five components, which were at 1615, 1638, 1655, 1670 and 1687 cm^-1^, respectively. These five components were all corresponding to the different secondary structure [17]. The two bands at 1638 and 1687 cm^-1^ were the amide groups involved in the extended *β*-sheet structure, while the band at 1655 cm^-1^ arose from either the *α*-helix or random coil structures. The 1670 cm^-1^ component can be due to the presence of *β*-turns and the weak shoulder at 1615 cm^-1^ resulted from intermolecular *β*-sheets due to protein aggregation. The content of the protein secondary structure segments of the amide I bands fitted can be calculated by ratios to the area of the amide I bands [16], which was used to investigate the protein secondary structure of amid I bands in the infra-red and Raman spectrum [18,19].

**Figure 4:**
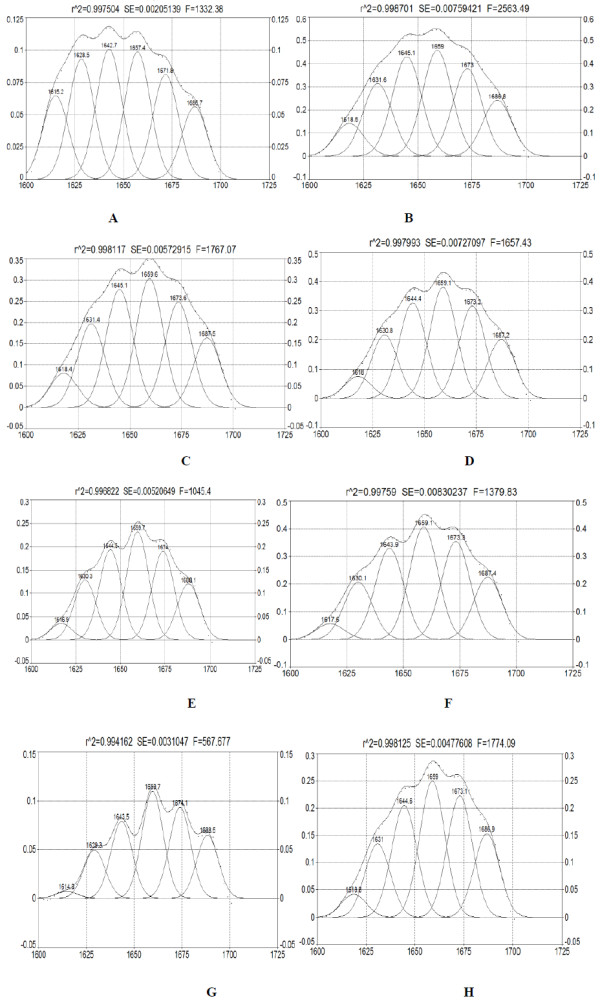
Deconvoluted infrared amide I bands of casein and casein hydrolysates (A-H, casein hydrolysate obtained after 0, 10, 20, 30,40, 60,80 and 100 min of hydrolysis).

Deconvolution curves of the amide I infrared absorption bands of original casein and freeze-dried casein hydrolysate samples were shown in Figure 4. Change in secondary structure of casein and casein hydrolysates obtained by FTIR spectrum and deconvolution curves were listed in Table 3. Casein protein contained 19.95% α-helix, 24.78% β-turn, 48.8% β-sheet and 20.38% random coil. No obvious changes in content of random coil were found for casein sample and casein hydrolysates. Increase in the contents of a-helix and β-turn in casein hydrolysates was noticed compared to the original casein sample. Moreover, there were increase trend for the contents of a-helix and β-turn with enzymatic hydrolysis. In addition, the contents of β-sheet in casein hydrolysates decreased with enzymatic hydrolysis. The content of β-sheet in the casein hydrolysate obtained from enzymatic hydrolysis for 100 min decreased to 17.44% from 31.89% of original casein. The molecular flexibilities of the casein hydrolysates (0.73 ~ 0.66), estimated by the ratio of α-helix to β-structure, were lower than that of original casein protein (0.81) (Table 3). This possibly caused by the fact that the enzymatic hydrolysis reduced the size of casein molecules by rupturing of the polypeptide chains. The enzyme action point was just the amino acid residue that formed the *α*-helix and *β*-turn and *β*-sheet.

**Table 3:** Change in secondary structure of casein and casein hydrolysates

**Hydrolysis time (min)**	***α*-helix(%)**	***β*-sheet (%)**	***β*-turn(%)**	**Random coil (%)**	**α-helix/β-staucture**
casein	19.95	31.89	24.78	20.38	0.81
10	23.29	23.23	31.60	21.88	0.73
20	23.87	21.88	32.37	21.88	0.73
30	25.03	19.34	34.15	21.49	0.73
40	25.76	18.23	34.48	21.52	0.74
60	25.73	16.76	36.68	20.83	0.70
80	27.18	14.04	39.22	19.57	0.69
100	24.83	17.44	37.36	20.37	0.66

### SDS-PAGE profile

**Figure 5:**
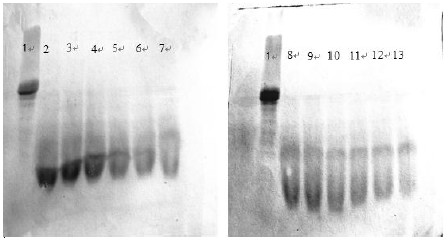
SDS-PAGE pattern of casein hydrolysates (1, casein, 2–13, hydrolysate obtained after 10, 20, 30, 40, 60, 80 and 100 min of hydrolysis).

SDS-PAGE pattern of the casein and casein hydrolysates is shown in Figure 5. Obviously, the numbers of protein bands of casein hydrolysates increased compared to the original casein sample (Lane 1) in SDS-PAGE pattern. Especially, the band numbers in the region with lower molecular weight began to rise gradually with increasing enzymatic hydrolyzation. It indicated the peptides with higher molecular weight broke up during enzymatic hydrolysis.

## Conclusions

The molecular weight distribution of casein hydrolysates obtained after enzymatic hydrolysis in casein-trypsin system changed significantly with increasing enzymatic hydrolyzation. Increase in the contents of a-helix and β-turn in casein hydrolysates was found compared to the original casein sample. Moreover, there were increase trend for the contents of a-helix and β-turn with enzymatic hydrolysis. In addition, the contents of β-sheet in casein hydrolysates decreased with enzymatic hydrolysis. The molecular flexibilities of the casein hydrolysates, estimated by the ratio of α-helix to β-structure, were lower than that of original casein protein.

## Competing interests

The authors declare that they have no competing interests.

## Authors’ contributions

JW made a significant contribution to experimental design, data analysis and manuscript preparation. YS mainly participated in the experiments. FJ made a substantial contribution to experimental design and data analysis. HJ participated in the design of the study and some of experiments. All authors read and approved the final version of the manuscript.

## References

[B1] Abu DO Bani-Jaber A Amro B Jones D Andrews GP The manufacture and characterization of casein films as novel tablet coatings Food and Bioproducts Process 2007 85 284 290 10.1205/fbp07030

[B2] Bryant CM McClements DJ Molecular basis of protein functionality with special consideration of cold-set gels derived from heat-denatured whey Trends in Food Scicence & Technology 1998 9 143 151 10.1016/S0924-2244(98)00031-4

[B3] Frisher H Meisel H Schlimme E OPA method modified by use of N, N-dimethyl-2-mercaptoethylammonium chloride as thiol components Fresenius Z Analytical Chemistry 2011 330 631 633

[B4] Liu Y Guo R pH-dependent structures and properties of casein micelles Biophys Chem 2008 136 67 73 10.1016/j.bpc.2008.03.012 18583019

[B5] Swaisgood HE Fox PF, Sweeney PLH Chemistry of the caseins Advanced dairy chemistry—1, proteins 2003 3 rd New York: Kluwer Academic/Plenum 139 201 Part A

[B6] Mocanua AM Moldoveanub C Lucia Odochianb L Cristina MP Apostolescua N Neculauc R Study on the thermal behavior of casein under nitrogen and air atmosphere by means of the TG-FTIR technique Thermochim Acta 2012 546 120 126

[B7] Holt C Fox PF The milk salts and their interaction with casein Advanced dairy chemistry 1997 London: Chapman & Hall 233 256

[B8] Damodaran S Hettiarachchy NS, Ziegler GR Structure-function relationship of food proteins Protein functionality in food systems 1994 New-York: Marcel Dekker 1 37

[B9] Kinsella JE Milk proteins: physicochemical and functional properties Crit Rev Food Sci Nutr 1984 21 197 262 10.1080/10408398409527401 6391823

[B10] Corredig M Dalgleish DG Studies on the susceptibility of membrane-derived proteins to proteolysis as related to changes in their emulsifying properties Food Res Int 1997 30 9 689 697 10.1016/S0963-9969(98)00018-0

[B11] Frisher H Meisel H Schlimme E OPA method modified by use of N, N-dimethyl-2-mercaptoethylammonium chloride as thiol components Fresenius Zeitschrift Analytical Chemistry 1988 330 631 633 10.1007/BF00473782

[B12] Bildlngmeyer BA Cohen SA Tarvin TL Frost B A new, rapid, high sensitivity analysis of amino acid in food type samples Journal of American Oil Chemistics Society 1987 70 241 247 3571118

[B13] Laemmli UK Cleavage of structural proteins during the assembly of the head of bacteriophage T4 Nature 1970 227 680 685 10.1038/227680a0 5432063

[B14] Deeslie WD Cheryan M Fractionation of soy protein hydrolysates using ultrafiltration memebranes J Food Sci 1991 57 411 413

[B15] Arrondo JLR Muga A Castersana J Quantitative studies of the structure of proteins in solution by Fourier-transform infrared spectroscopy Prog Biophys Mol Biol 1993 59 23 56 10.1016/0079-6107(93)90006-6 8419985

[B16] Surewicz WK Mantsch HH New insight into protein secondary structure from resolution-enhanced infrared spectra Biochim Biophys Acta 1988 952 115 130 3276352 10.1016/0167-4838(88)90107-0

[B17] Subirade M Kelly I Guéguen J Molecular basis of film formation from a soybean protein: comparison between the conformation of glycinin in aqueous solution and in films Int J Biol Macromol 1998 23 241 249 10.1016/S0141-8130(98)00052-X 9849621

[B18] Dousseau F Pézolet M Determination of the secondary structure content of proteins in aqueous solutions from their amide I and amide II infrared bands. Comparison between classical and partial least-squares methods Biochemistry 1990 29 37 8771 8779 10.1021/bi00489a038 2271555

[B19] Lee DC Haris PI Chapman D Determination of protein secondary structure using factor analysis of infrared spectra Biochemistry 1990 29 9185 9193 10.1021/bi00491a012 2271587

